# A study on the epidemiological characteristics and infectious forecast model of malaria at Guangzhou Airport among Chinese returnees from Africa

**DOI:** 10.1186/s12936-017-1927-4

**Published:** 2017-07-04

**Authors:** Hui-ming Wu, Zhi-qiang Fang, Dang Zhao, Yan-ling Chen, Chuan-ge Liu, Xi Liang

**Affiliations:** 1 Guangzhou Airport Entry-Exit Inspection and Quarantine Bureau, Guangzhou, 510470 China; 2 Chinese Academy of Inspection and Quarantine, Beijing, 100176 China

**Keywords:** Chinese travellers, Africa, Malaria, Epidemiological characteristics, Forecast model

## Abstract

**Background:**

Cross-border malaria transmission in China is a major component of Chinese imported malaria cases. Such cases mostly are travellers returning from malaria endemic countries in Africa. By investigating malaria infectious status among Chinese worker in Africa, this study analysed the malaria risk factors, in order to establish infectious forecast model.

**Methods:**

Chinese returnees data from Africa were collected at Guangzhou Baiyun International Airport, Guangzhou, China between August 2015 and March 2016 and were included in the cross-sectional and retrospective survey.

**Results:**

A total of 1492 respondents were included in the study with the majority consisting of junior middle school educated male. Most of them are manual and technical workers hired by companies, with average of 37.04 years of age. Overall malaria incidence rate of the population was 8.98% (134/1492), and there were no significant differences regarding age, gender, occupation, or team. Forecast model was developed on the basis of malaria risk factors including working country, local ecological environment type, work duration and intensity of mosquito bite prevention.

**Conclusions:**

The survey suggested that malaria incidence was high among Chinese travellers who had worked in Africa countries of heavy malaria burden. Further research on the frequency and severity of clinical episodes among Chinese travellers having worked in Africa is needed.

## Background

Malaria is one of the most common and serious life-threatening tropical diseases worldwide. In 2015, an estimated 3.2 billion people (half of the world’s population) were at risk of malaria, and most infections and deaths occurred in sub-Saharan Africa [1]. Imported malaria cases from Africa is becoming a threat to many countries [2, 3]. In 2014, 3022 imported malaria cases were reported in China, which accounted for 98.2% (3022/3078) of the total cases, while the returnees from malaria-endemic countries in Africa accounted for almost 75% of the imported malaria cases. Moreover, the top 5 countries with the most imported malaria cases were Myanmar, Nigeria, Equatorial Guinea, Angola and Ghana, respectively [4]. In 2013, 65% of all 1501 malaria cases reported in UK were acquired in West Africa, nearly 80% were by *Plasmodium falciparum* and 82% were among people who were visiting friends and relatives (VFRs) [5]. From 2006 to 2014 a total of 185 patients with severe malaria treated in 12 European countries were included in an 8-year multi-center observational study while the majority of infections were acquired in West Africa (109/185) [6].

Every year, approximately one million Chinese travellers without immunity work in Africa and are susceptible to malaria. During May–August 2013, a malaria outbreak comprising 874 persons in Shanglin County located in Guangxi province in China, was detected among 4052 people returning from overseas. Ghana was the predominant destination country, and 92.3% of malaria infections occurred in gold miners [7]. Thus imported malaria cases from Africa is a new challenge for malaria elimination in China [8–14]. Although remarkable progress has been made in reducing the global malaria burden, this disease remains endemic in many regions, and appropriate prevention measures by travellers is still inadequate. There is a lack of information regarding epidemiology of the imported malaria cases among Chinese returnees worked in Africa. Therefore, the objective of this study was to investigate the malaria infectious status among Chinese returnees from Africa and infectious risk factors.

## Methods

### Inclusion criteria

The survey was carried out for epidemiological investigation among Chinese returnees at Guangzhou Baiyun International Airport from August 2015 to March 2016. Chinese returnees who had worked in Africa no less than 1 month were interviewed, including manual labour, businessmen and businesswomen, and technical workers, but excluding tourists, or students. Two kinds of questionnaires were distributed among participants, depending on the infectious history.

The first questionnaire was completed by all participants for the following information:

- Demographic details;
- Working history;
- Malaria chemoprophylaxis used.

The second questionnaire including the following information were designed for participants who experienced malaria infection:

- Infectious history;
- Exposure history;
- Treatment details.

### Data collection

A random sampling was conducted to select returnees working in Africa (Chinese worker in Africa) at Guangzhou Baiyun airport quarantine site according to the appearance, clothing, language and luggage. They were asked to participate the survey by trained interviewers. Blood was took from febrile returnees and the volunteers for rapid diagnostic test, before stored at 4 °C for further laboratory test within 24 h. Respondents with temperature above 37.5 °C were considered as “febrile on examination” and these febrile respondents would be follow up in 1 month for identification. Blood smear microscopy was performed in the laboratory certified by CNAS (China National Accreditation Service for Conformity Assessment).

### Malaria diagnosis

The diagnostic criteria (WS259-2015) for malaria was adopted in this survey. Febrile returnees detected on site, with positive blood test results, would be considered malaria cases. Returnees with malaria medical record could be defined as malaria cases.

### Data analysis

All analysis was performed using SPSS 16.0 software (IBM, Armonk, US). The descriptive analysis and Chi squares were used and the logistic regression was conducted in order to build the forecast model. The significance level was set at 0.05.

## Results

A total of 1507 questionnaires were collected, 1492 of which were valid and were included in the analysis with an effective rate of 99% in this study. There are 134 respondents considered as malaria cases in this survey. Another 128 respondents without fever confirmed that they had been infected with malaria in Africa, and 6 respondents with fever were diagnosed with malaria. In total, 65 blood samples were taken, 11 of which were positive for malaria while 10 blood samples were found to be *Plasmodium falciparum* and one was confirmed to be *Plasmodium vivax* with blood smear microscopy. However, 5 of the respondents experienced malaria infection history in Africa and the other 6 patients refused to admit previous malaria infection. Therefore, this malaria incidence rate among the returned travellers from Africa was 8.98% (134/1492).

### Analysis of epidemiological characteristics

#### Gender analysis

Overall, 1364 respondents were male and 128 were female. The sex ratio was 10.66:1. Incidence was not significantly associated with gender ($\chi^{ 2} = 2. 1 1 3$, *P* = 0.146 > 0.05). The result is shown in Table 1.

**Table 1:** Analysis of the epidemiological characteristics and infectious status of Chinese worker in Africa

Variable	Number (ratio %)	Malaria positive (incidence rate %)	$\chi^{ 2}$	*P*
Gender			2.113	0.146
Male	1364 (91.4)	127 (9.31)		
Female	128 (8.6)	7 (5.47)		
Age (years)			0.988	0.912
16	156 (10.5)	14 (8.97)		
26	563 (37.7)	49 (8.70)		
36	441 (29.6)	41 (9.30)		
46	303 (20.3)	26 (8.58)		
56–65	29 (1.9)	4 (13.79)		
Educational level			8.885	0.003
Senior high school below	621 (41.62)	72 (11.59)		
Senior high school and above	871 (58.38)	62 (7.12)		
Profession			0.524	0.769
Manual labor	769 (51.5)	71 (9.23)		
Technical worker	455 (30.5)	42 (9.23)		
Business	268 (18.0)	21 (7.84)		
Team types			0.324	0.850
Private company	849 (56.9)	75 (8.83)		
State-owned company	528 (35.4)	47 (8.90)		
Others	115 (7.7)	12 (10.43)		

#### Age analysis

The minimum age was 18 years old, and maximum age 63 years old, with an average a of 37.04 ± 0.24 years of age. Incidence was not significantly associated with age ($\chi^{ 2} = 0. 9 8 8$, *P* = 0.912 > 0.05).

#### Education analysis

The majority of respondents were junior high school education level (35.39%), with 41.62% of them did not finish senior high school. Incidence of the senior high school and the below was significantly higher than senior high school and the above ($\chi^{ 2} = 8. 8 8 5$, *P* = 0.003 < 0.05).

#### Profession analysis

Of 1492 respondents, 51.5% was manual labor, 30.5% technical workers and 18.0% business. Incidence was not significantly associated with profession ($\chi^{ 2} = 0. 5 2 4$, *P* = 0.769 > 0.05).

#### Team analysis

Respondents from private companies accounted for 56.9% of the total, with 35.4% from state-owned companies, and 7.7% from other team types. Incidence was not significantly associated with team types ($\chi^{ 2} = 0. 3 2 4$, *P* = 0.850 > 0.05).

### Analysis of working countries and local environment

#### Working countries analysis

**Fig. 1:**
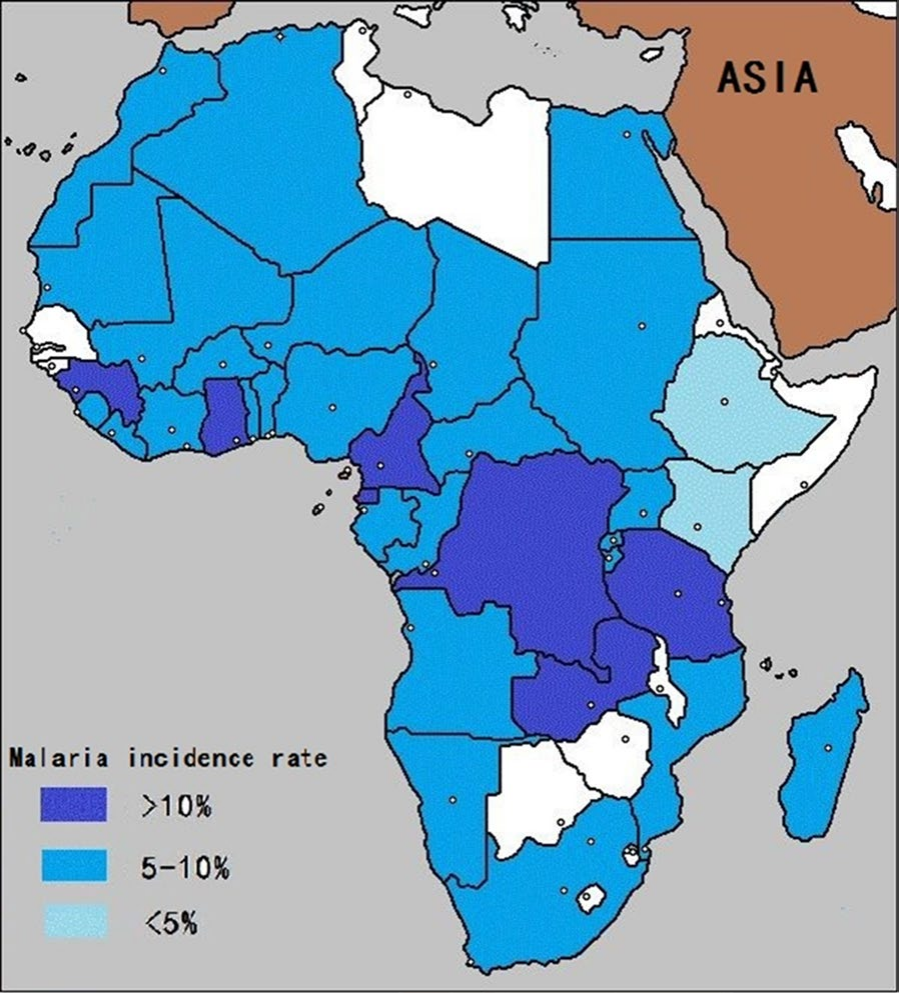
Malaria infectious status of Chinese workers in Africa

The 1492 respondents returned from 41 countries in Africa consisting 56 countries. There are 233 Chinese (31.2%) worked in Ethiopia and Congo respectively, which were the biggest proportion in Africa. According the survey, 134 malaria cases came from 22 countries. The top 7 countries with higher malaria incidence rate were Ghana (24.59%), Cameroon (22.73%), Equatorial Guinea (22.58%), Zambia (17.65%), Guinea (15.79%), Democratic Republic of the Congo (11.24%) and Tanzania (10.26%). Apparently, incidence was significantly associated with countries ($\chi^{ 2} = 6 6. 8 6 8$, *P* = 0.000 < 0.05). The result is shown in Fig. 1.

#### Local ecological environment

Plasmodium was mostly transmitted by anopheles mosquitoes, thus the environment is a determinant in malaria transmission. Five types of the local ecological environment includes city, island, plain, mountain and others. Incidence was significantly associated with environment ($\chi^{ 2} = 2 5. 8 8 9$, *P* = 0.000 < 0.05). There were 6.88% Chinese workers lived in islands, city and plain had infected with malaria, while 15.6% Chinese workers lived in mountain and other experienced infection. The results are shown in Table 2.

**Table 2:** Analysis of the infectious status of Chinese worker in Africa at different ecological environment

Variable	Number	Infection rate (%)	$\chi^{ 2}$	*P*
Types of environment			25.889	0.000
Island	68	3 (4.41)		
Plain	219	15 (6.85)		
City	846	60 (7.10)		
Mountain	313	49 (15.65)		
Others	46	7 (15.22)		

### Analysis of working duration

Overall, 35.92% (536/1492) respondents had worked in Africa from half 6–18 months. The probability of infection would increase with the work duration ranged from 1 to 24 months. Whereas, the probability became stable or decreased after 24 months. The result is shown in Table 3.

**Table 3:** Analysis of the correlation between work duration and infectious status

Working duration (months)	Number	Malaria positive (incidence rate %)	$\chi^{ 2}$	*P*
1–3	270	8 (2.96)	25.380	0.001
4–6	366	27 (7.38)		
7–9	183	13 (7.10)		
10–12	353	42 (11.90)		
13–15	99	13 (13.13)		
16–18	71	10 (14.08)		
19–21	21	3 (14.29)		
22–24	77	12 (15.58)		
Above 24	52	6 (11.54)		

### Analysis on the compliance with recommended preventive measures

**Fig. 2:**
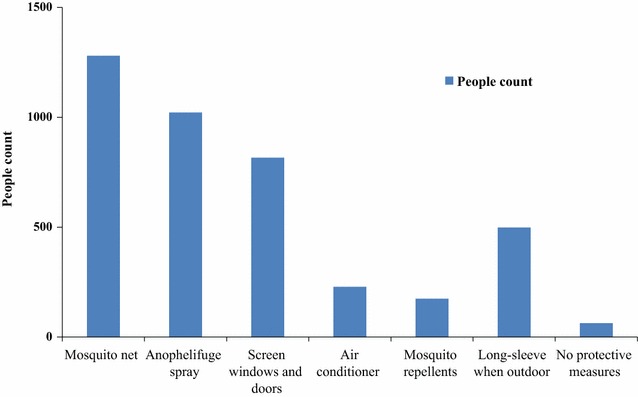
Analysis on the compliance with recommended preventive measures

Protecting from mosquito bite is an important strategy to prevent malaria. The adoption rate of several anti-mosquito measures was: mosquito net 85.79% (1280/1492), anophelifuge spray 68.50% (1022/1492), screen windows and doors 54.69% (816/1492), wearing long-sleeve clothes during outdoor activities 33.38% (488/1492), air conditioning 1.88% (28/1492), mosquito repellents on exposed skin 11.66% (174/1492). However, 4.22% (63/1492) cases did not take any protective measures in the survey (Fig. 2).

**Fig. 3:**
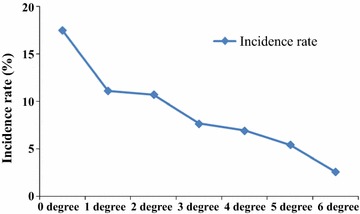
Analysis of the correlation between intensity of mosquito bite prevention and malaria infection

Some of these 6 measures were adopted at the same time to help individuals prevent malaria. All the 1492 respondents were grouped with the number of protective measures adoption as intensity of mosquito bite prevention: group without any protective measure were defined as “0 degree”; group adopting one measure were defined as “1 degree”; group adopting two measures were defined as “2 degree”, and so on. Data showed that malaria incidence rates decreased with the rising of intensity of mosquito bite prevention (Fig. 3).

### Study on risk factors and forecast model of malaria among Chinese worker in Africa

The malaria infectious risk factors analysed by mono-factor analysis method were: education background, working country, local ecological environment type, work duration and intensity of mosquito bite prevention. Dependent variable showed whether those cases were infected with malaria, 1 = infected, 0 = uninfected (Table 4). According to the malaria prevalence of the different countries in World Malaria Report 2015, these countries were divided into three groups: countries with incidence higher than 20%, called high-endemic countries, including Zambia, Uganda, Tanzania, Ghana and Angola; countries with incidence between 10 and 20%, called middle-endemic countries, which included Mozambique, Cameroon, Gabon, the Republic of Congo, Guinea and Nigeria; countries with incidence between 5 and 10%, called low-endemic countries, which included Congo, Ethiopia and Equatorial Guinea.

**Table 4:** Variable assignments of multivariate unconditioned logistic regression

Factors	Assignments
Educational background (X_1_)	1 = High school degree or above, 2 = below high school degree
Working countries (X_2_)	Countries were divided into three groups according the incidence, 3 = >20% (high-affected courtiers), 2 = 10–20% (middle-affected countries), 1 = 5–10% (low-affected countries)
Type of the local ecological environment (X_3_)	According the infection rate, types separated into two groups, 2 = mountainous regions and others, 1 = city, plain and island
Working duration (X_4_)	Figured on a monthly basis
Intensity of mosquito bite prevention (X_5_)	Cases without any protective measure are defined as a set of “0”; cases using one measure are defined as a set of “1”; cases using two measures are defined as a set of “2”, and so on

Data were analysed with SPSS16.0 software. The best regression model of infectious forecast situation is as follows:

$${\text{P}}\left( 1 \right){ = 1/}\left[ { 1 {\text{ + e}}^{{\, -\, \left( { 4. 8 9 3\,-\, 0. 6 1 6 {\text{X}}_{ 2}\, -\,0. 9 2 7 {\text{X}}_{ 3} \,-\, 0. 0 2 8 {\text{X}}_{ 4}\, +\, 0. 2 5 8 {\text{X}}_{ 5} } \right)}} } \right]$$

P (1) means the malaria incidence rate of Chinese worker in Africa.

In this regression model, the estimation of parameter and OR value are indicated in Table 5.

**Table 5:** Result of multivariate unconditioned logistic regression on infectious risk factors

Variable	Regression coefficient B	Standard deviation SE	Wald $\chi^{ 2}$ value	*P* value	OR value	95% CI	
						Floor	Ceiling
Constant	4.893	0.502	94.802	0.000			
X_2_	−0.616	0.123	24.900	0.000	0.540	0.424	0.688
X_3_	−0.927	0.199	21.709	0.000	0.396	0.268	0.585
X_4_	−0.028	0.009	10.649	0.001	0.972	0.955	0.989
X_5_	0.258	0.072	12.970	0.000	1.295	1.125	1.490

## Discussion

The results of the survey demonstrated that overall malaria incidence rate of Chinese workers in Africa was 8.98% at high risk and many respondents confirmed that they had experienced malaria infection more than once. The top five countries with higher malaria incidence were Ghana, Cameroon, Equatorial Guinea, Zambia and Guinea which are high malaria burden countries [1]. Consequently, malaria prevalence in African countries plays an important role on the malaria infection for Chinese travellers.

Eleven of 65 blood samples in this survey were positive for malaria, while 6 of 11 patients denied that they had previous malaria infection history in Africa. When the imported malaria cases and travellers with the asymptomatic malaria infection return from Africa, it could presented as a serious threat to Chinese malaria control [2, 4, 8]. Malaria infections can be fatal if not diagnosed and treated promptly with anti-malarial medications appropriate for the patient’s age and medical history [15]. However, it is difficult for those imported malaria patients to seek timely medical help since there are so rare indigenous malaria cases that the doctors may not be familiar with malaria and the anti-malarial medicines are seldom stored in Chinese hospitals [4, 9].

The public awareness of malaria in China needs continuously improvement [16]. One survey showed high level of ignorance among Chinese international travellers for the need of seeking pre-travel medical advice and travel health preparedness [17]. Moreover, this survey showed that there was low compliance with recommended preventive strategies among the group, and 4.22% (63/1492) of the total respondents did not take any preventive strategies in the survey. Chinese travellers usually take medicine for malaria treatment instead of prevention and they consider such way to be easier. It is not convenient for Chinese travellers to take chemoprophylaxis in Africa for a long time.

The forecast model among Chinese travellers who had worked in Africa is based on malaria risk factors which suggested that educational background was not included and the malaria risk factors are complicated. While it may be important to increase the awareness of the potentially fatal consequences of malaria among travellers who worked in Africa, many studies have demonstrated that there is no direct linkage between knowledge and behavior. Health behaviours are grounded in complex socio-ecological context where there are frequently structural and social barriers constraining actions [18].

Altering the behavior of travellers who had worked in Africa and reducing the malaria burden requires more than a didactic knowledge transferring approach as is currently advocated. Alternative policy should consider a focus on solving practical issues, including self-management of malaria, early diagnosis and rapid treatment through primary and urgent care centers and easy access to effective malaria treatments [19–21]. Epidemiological characters of the survey suggested that a more effective strategy for the high-risk group should be developed. Additional epidemiological data on the frequency and severity of clinical episodes among Chinese travellers who had worked in Africa is needed.

One of the study limitations was that some travellers deliberately concealed the malaria infection history so that the true malaria incidence was probably higher than the indicated number in the current survey. Secondly, the majority respondents who worked in Africa with poor medical level so that they usually just took the medicine with a lack of the laboratory test because malaria is so epidemic that they simply considered the condition as cold. In addition, there might also be asymptomatic malaria infections.

## Conclusions

The epidemiological evidence suggested that malaria incidence among Chinese travellers who worked in Africa was high and the current guidelines for the imported malaria prevention in China are not working effectively in this specific group. Reasons for this epidemiological consequence includes Chinese health beliefs held by this population as well as structural, social, environmental, and economic context within which malaria prevention decisions are made. In order to develop more effective strategies to reduce the malaria burden among Chinese travellers worked in Africa, incorporating this evidence in the survey may be helpful. The authors recommend that for people going to work in Africa: the first step should be getting the pre-travel advice and check the destination country whether it is a high malaria rate county; secondly, the person should take high compliance with the preventive measures.

## Abbreviations

VFRs: visiting friends and relatives
RDT: rapid diagnostic test
CNAS: China National Accreditation Service for Conformity Assessment
